# Development and validation of mortality risk prediction model for sepsis secondary to pneumonia at intensive care unit admission: a retrospective case-cohort study

**DOI:** 10.3389/fmed.2025.1592325

**Published:** 2025-08-12

**Authors:** Xiaoliang Li, Xiaoli Lei, Lijie Kou, Junli Wang, Zhigang Yang

**Affiliations:** ^1^Department of Respiratory and Critical Care Medicine, Henan Provincial People's Hospital, Zhengzhou, China; ^2^Department of Cardiopulmonary Function, Fuwai Central China Cardiovascular Hospital, Zhengzhou, China

**Keywords:** sepsis, pneumonia, SOFA score, APACHE II score, mortality

## Abstract

**Background:**

To establish a noninvasive mortality risk prediction model for sepsis secondary to pneumonia (SSP) and validate the model in the prediction of mortality risk in SSP patients at hospital admission.

**Methods:**

A retrospective cohort of SSP patients were recruited from January 2017 to December 2020 at the Henan Provincial People’s Hospital. Clinical data were collected at admission. Least absolute shrinkage and selection operator and logistic regression were used to construct a prognosis prediction model. The predictive performance of the model was evaluated by receiver operating characteristic (ROC) curve. Another retrospective cohort with SSP was recruited from January 2021 to July 2022 at the same hospital to validate the model.

**Results:**

A total of 1,337 patients were screened, including 941 patients in the derivation cohort and 396 patients in the validation cohort. The model included age, white blood cell count, neutrophil-to-lymphocyte ratio, lactate dehydrogenase, arterial oxygen pressure / fraction inspired oxygen, D-dimer and vasoactive drug use. The area under ROC curve of derivation model was better than sequential organ failure assessment score and APACHE II score (0.777 *vs.* 0.600 *vs.* 0.625, *p* < 0.05). Besides, the proposed model had a significantly higher prediction performance than SOFA and APACHE II scores in the validation cohort (0.803 *vs.* 0.655 *vs.* 0.688, *p* < 0.05). The prediction model was publicly released as an online calculator.

**Conclusion:**

A prognosis model based on variables of SSP patients at hospital admission was developed.

## Introduction

Pneumonia is responsible for great mortality and high costs. The World Health Organization reports around 2.5 million population died of lower respiratory infection mainly caused by pneumonia, making it the fourth cause of death globally and also the most deadly infectious disease (1). According to the China Public Health Statistical Yearbook 2019, the prevalence rate of pneumonia is 1.1‰ in 2008 and 0.6‰ in 2013, while the mortality rate is 17.46 and 15.09 per 100 thousand in 2012 and 2018, respectively (2). Sepsis is the organ dysfunction with a high mortality rate due to dysregulated inflammatory response in the host (3). In 2017, there were roughly 4.89 million sepsis patients around the globe, 1.10 million of whom died of sepsis, accounting for 19.7% of global death (4). In China, one fifth sepsis patients are admitted into ICU with a 90-day mortality rate of 35.5%, and the leading cause of sepsis is pneumonia (5). Sepsis secondary to pneumonia (SSP) is characterized by the lack of specific clinical manifestations during the early stage, but it progresses rapidly with poor prognosis. Therefore, early prediction of SSP mortality risk helps to correctly identify high-risk patients and timely adjust treatments, which may be crucial for improving the patients’ outcomes and survival rate.

Sepsis has complex pathogenetic mechanisms involving multiple systems including systemic inflammatory response, oxidative stress, coagulation system and immune system (6). Thus combined detection may be useful for improving the predictive accuracy. Several scoring systems have been proposed for prognosis assessment of pneumonia and sepsis, but the sensitivity and specificity are not consistent in published studies (7–11). Sequential organ failure assessment (SOFA) was proposed by European Society of Intensive Care Medicine in 1994 (12). According to the publication of Sepsis 3, organ dysfunction can be represented by an increase in the SOFA score of 2 points or more, which is associated with an in-hospital mortality. Pawar et al. (13) found that the predict mortality of SOFA score is different substantially based on infection type location. Besides, the SOFA score, taking no account comorbidities, age and physiologic status, performed lower than the other score (14). The score of CURB-65 including confusion, urea, respiratory rate, blood pressure, age ≥ 65 years is not effective for hospitalized patients mortality predication (15). Kolditz et al. (16) found that CURB-65 score is poor for identifying severe community acquired pneumonia (CAP) patients. It might because that the CURB-65 does not incorporate hypoxaemia and bilateral pneumonia in their scores (17). Similarly limitation also exists in APACHEII score that it consists of 20 variables, which are usually unavailable within 24 h after admission to hospital. So Ferrer reported that PSI, CURB-65 or acute physiology and chronic health evaluation II (APACHE II) score does not identify the severe CAP patients with a relatively high mortality risk (18). This study aims to identify the risk factors related to 28-day mortality in SSP patients and to develop and validate a multi-parameter combined prediction model.

## Methods

### Data collection and management

In this retrospective, single-center study, we collected data from 1,337 SSP patients admitted into respiratory ICU between January 2017 and June 2022. The patients were divided into model derivation cohort (January 2017 to December 2020) and validation cohort (January 2021 to June 2022). In this study, we defined study outcome as 28-day death and divided the patients into either survival group or death group. This study was approved by the Henan Provincial People’s Hospital Ethics Committee. Written informed consent was waived due to the retrospective use of patients’ data.

CAP and hospital acquired pneumonia (HAP) were diagnosed according to the guidelines jointly published by Infectious Diseases society of America and America Thoracic Society in 2019 and in 2016, respectively (19, 20). CAP was diagnosed if the patient had pulmonary infiltrates on chest imaging examination and presented one of the following clinical presentations: (1) cough, expectoration, (2) fever, (3) white blood cell count > 10 × 10^9^/L or < 4 × 10^9^/L, or (4) physical examination indicating pulmonary consolidation signs. HAP was diagnosed if the patient had new or progressive pulmonary infiltrates 48 h after admission and one of the following infectious manifestations: (1) fever, (2) purulent sputum, or (3) elevated white blood cell count. Sepsis was diagnosed if infection and sepsis-related SOFA score ≥ 2 according to The Third International Consensus Definitions for Sepsis and Septic Shock (Sepsis-3) published in 2016 (3).

All data were evaluated, extracted and cross-validated by a group of experienced respiratory physicians. Each recording was independently checked by two physicians. We included all the patients with the required clinical data (laboratory tests, clinical symptoms and signs, severity assessment and discharge information) during hospitalization. The patients were followed up for 28 days.

### Inclusion and exclusion criteria

Inclusion criteria: (1) age ≥ 18 years, (2) the patient was diagnosed with both pneumonia and sepsis, and (3) the patient had all the required clinical data.

Exclusion criteria: (1) age < 18 years, (2) history of malignant tumor in the past 5 years, (3) human immunodeficiency virus infection, (4) any connective tissue disease such as rheumatoid arthritis, systemic lupus erythematosus or vasculitis, (5) drug therapy with hormone, immunosuppressor or immunoregulation drug in the past 3 months, (6) organ transplantation, (7) in-hospital death within 24 h of admission; and (8) the patient having incomplete clinical data.

### Potential predictive variables

The following characteristics at hospital admission were included as potential predictive variables: demographic data, medical history, physical signs and laboratory tests. Demographic data included age, sex, history of smoking, and history of drinking. Medical history included chronic obstructive pulmonary disease, diabetes mellitus, hypertension, coronary artery disease, cerebrovascular disease and so on. Physical signs and symptoms included body temperature, respiratory rate, heart rate, blood pressure and state of consciousness. Laboratory tests included white blood cell count, neutrophil count, lymphocyte count, platelet count, neutrophil-to-lymphocyte ratio (NLR), hemoglobin, C-reactive protein (CRP), procalcitonin (PCT), lactic dehydrogenase, aspartate transaminase, alanine transaminase, total bilirubin, creatine kinase, creatinine, urea, total protein, albumin, blood sodium, blood potassium, D-dimer, prothrombin time, activated partial thromboplastin time, fibrinogen, fasting blood-glucose level, lactic acid and arterial blood gas test.

### Variable selection

The SSP patients in derivation cohort were used for variable selection and risk prediction model derivation. If the amount of missing values was less than 20%, the missing continuous values were replaced with median imputation technique. A total of 44 variables were finally selected. The least absolute shrinkage and selection operator (LASSO) regression was utilized to minimize the potential collinearity and overfitting of variables within a patient via the glmnet package in R software. Then, the variables selected from the LASSO regression were included in a logistic regression model, after which the statistically significant variables were used for constructing a mortality risk prediction model and for building a web-based risk calculator.

### Model derivation, external validation and evaluation of different models

The predictive performance of the proposed model was evaluated with ROC curve and calibration curve, and internally validated with reinforced bootstrap method. To evaluate the generalizability of the SSP risk prediction model, the patients admitted between January 2021 and June 2022 were used for external validation. The predictive performance of the proposed model in validation cohort was evaluated using discrimination and calibration.

The area under ROC curve (AUC) was utilized to compare the accuracy of our proposed model with those of SOFA and APACHE II.

### Statistical analysis

Categorical variables were shown as number and percentage (%) and compared using chi-squared test between groups. Normally distributed data were shown as mean ± standard and compared using t test between groups. Non-normally distributed data were shown as median (interquartile range) and compared using Mann–Whitney U test between groups. All statistical analyses were performed using R 3.6.2 (R Foundation) and SPSS 23.0 software (IBM). Statistical significance was defined as a *p* value < 0.05.

## Results

### Study population characteristics

**Figure 1:**
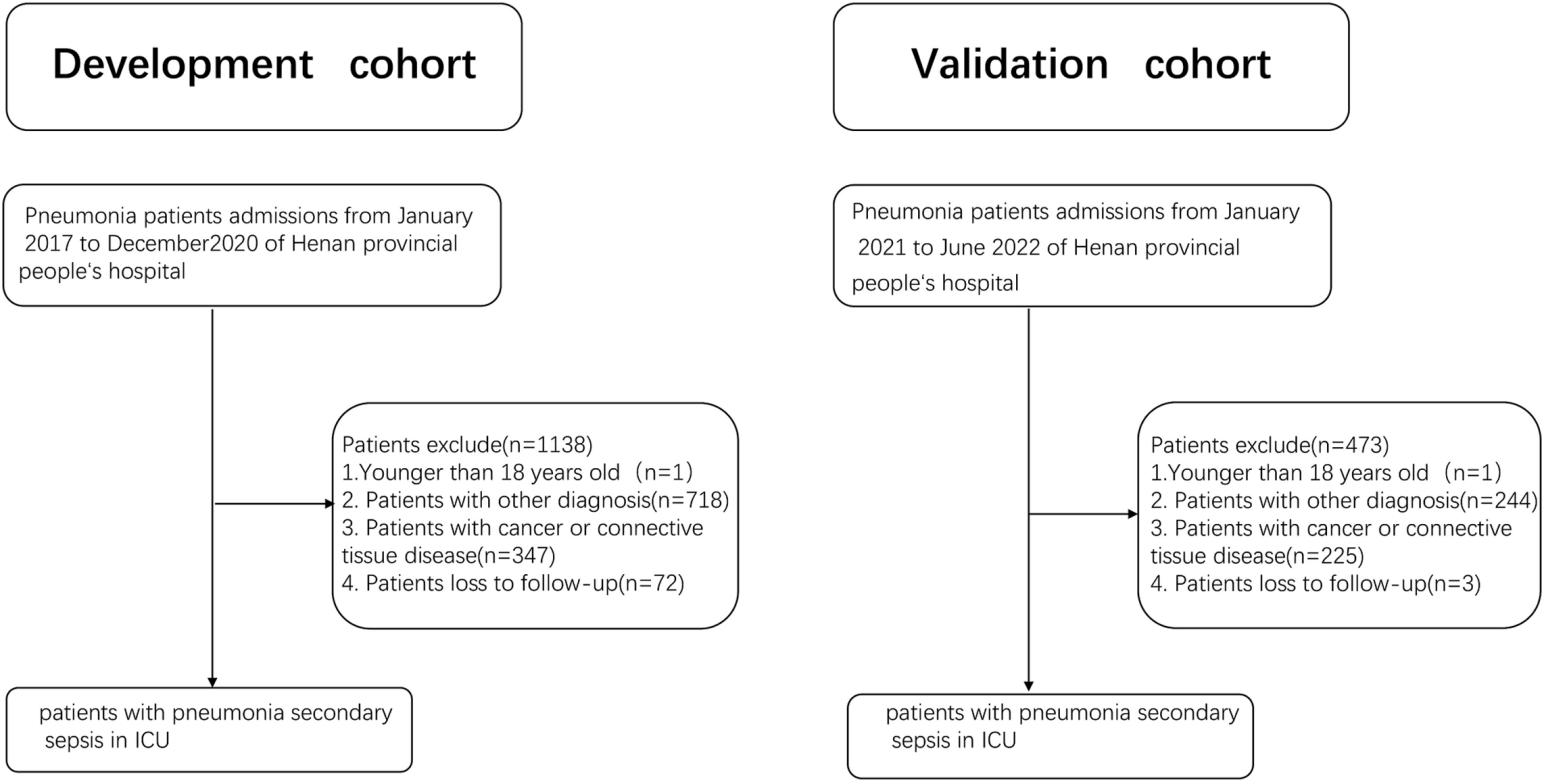
Patient flowchart of derivation and validation cohorts.

A total of 941 patients were included in the derivation cohort after excluding 1,138 pneumonia patients, with age younger than 16 years, complicated by tumor or connective tissue disease and lost to follow-up (Figure 1). The eligible patients had a median age of 71 years and were mainly male (*n* = 676, 71.8%). Mortality occurred in 461 (49.0%) patients. Most patients (89.0%) had at least one comorbidities, including cardiovascular disease (*n* = 651, 69.2%), cerebrovascular disease (*n* = 381, 40.5%) and diabetes (*n* = 231, 24.5%) (Table 1).

**Table 1:** Baseline characteristics of the two cohort.

Variables	Derivation cohort (*n* = 941)	Validation cohort (*n* = 396)
Age, years	71 (60–81)	70 (59–79)
Sex, *n* (%)		
Male	676 (71.8%)	292 (73.7%)
Female	265 (28.2%)	104 (26.3%)
Smoke	443 (47.1%)	178 (44.9%)
Drink	316 (33.6%)	125 (31.6)
Cardiovascular disease	651 (69.2%)	225 (56.8%)
Cerebrovascular disease	381 (40.5%)	173 (43.7%)
Diabetes	231 (24.5%)	117 (29.5%)
SOFA score	6 (4, 8)	6 (4, 8)
APACHE II score	18 (13, 22)	17 (12, 23)
Respiratory rate, cycles/min	23 (20, 29)	22 (20, 28.8)
Systolic blood pressure, mm Hg	122 (103.5, 142)	120 (103, 145)
Diastolic blood pressure, mm Hg	70 (60, 81)	68 (60, 78)
P/F, mm Hg	187.8 (131, 247.2)	203.0 (141.1, 272.6)
WBC count, ×10^9^/L	11.2 (7.5, 15.3)	11.5 (8.3, 16.5)
LYM count, ×10^9^/L	0.8 (0.5, 1.3)	0.7 (0.5, 1.1)
N/L	10.1 (6.1, 19.8)	13.7 (7.7, 24.0)
HgB, g/L	104 (87, 123)	106 (90, 120)
Platelet count, ×10^9^/L	188 (125.5, 267)	186 (123.2, 272.8)
TP, g/l	56.2 (51, 61.1)	56 (51.3, 60.1)
ALB, g/l	29.2 (26.2, 32.2)	30.2 (27.4, 33.6)
LDH, U/L	341 (245, 515.5)	321 (241.5, 466.2)
CRE, μmol/L	61 (45, 93)	63.5 (42, 108)
BUN, mmol/L	9 (6, 14.2)	10.0 (6.3, 15.1)
CRP, mg/L	98.8 (46.1, 163.2)	92.0 (42.3, 147.8)
PCT	0.8 (0.2, 3.4)	0.8 (0.2, 3.2)
Glu, mmol/L	7.1 (5,5, 9.7)	7.7 (6.3, 9.7)
D-dimmer, μg/mL	2.8 (1.5, 5.4)	3.1 (3.5, 6.5)
Lac, mmol/L	1.6 (1.2, 2.1)	1.6 (1.2, 2.2)
VD	403 (42.8%)	166 (42.0%)

P/F, PaO2/FiO2; WBC, white blood cell; LYM, lymphocyte; N/L, Neutrophils/lymphocyte; HgB, hemoglobin; TP, total protein; ALB, albumin; LDH, lactate dehydrogenase; CRE, creatinine; BUN, blood urea nitrogen; Glu, glucose,; Lac, lactate; VD, vasoactive drug.

Validation cohort included 396 patients (Figure 1). The eligible patients had a median age of 70 years and were mainly male (*n* = 292, 73.7%). Mortality occurred in 179 (45.2%) patients. Over half of the patients (57.1%) had at least one comorbidities, including cardiovascular disease (*n* = 225, 56.8%), cerebrovascular disease (*n* = 173, 43.7%) and diabetes (*n* = 117, 29.5%). The death group had a higher occurrence of septic shock (death group *vs.* survival group: 64.1% *vs.* 22.8%, *p* < 0.001) and a lower arterial oxygen pressure / fraction inspired oxygen (PaO2/FiO2) death group *vs.* survival group: 173.7 mm Hg (116.4–235.1) *vs.* 207 mm Hg (155.7–267.3), *p* < 0.001 (Table 2). Pathogens were detected in 651 patients, with gram-negative bacilli accounting for 49.43%, gram-positive cocci accounting for 11.49%, and fungi accounting for 13.79%.

**Table 2:** Clinical characteristics according to prognosis in the two cohorts.

Variables	Death		*p* value
	No (*n* = 697)	Yes (*n* = 640)	
Age, years	68 (56, 78)	74 (64, 82)	< 0.0001
Sex, *n* (%)			
Male	504 (72.3%)	464 (72.5%)	0.006
Female	193 (27.7%)	176 (27.5%)	
Smoke	307 (44.0%)	314 (49.1%)	0.066
Drink	234 (33.6%)	207 (32.3)	0.633
SOFA score	5 (3, 7)	7 (4, 9)	< 0.0001
APACHE II score	16 (11, 21)	19 (15, 24)	< 0.0001
Respiratory rate, cycles/min	23 (20, 29)	23 (20, 28)	0.302
Systolic blood pressure, mm Hg	118 (100, 148.5)	123 (107, 138.7)	0.086
Diastolic blood pressure, mm Hg	69 (58, 80.5)	70 (61, 80)	0.169
P/F, mm Hg	207 (155.7, 267.3)	173.7 (116.4, 235.1)	< 0.0001
WBC count, ×10^9^/L	10.2 (7.0, 13.8)	12.6 (8.8, 17.4)	< 0.0001
LYM count, ×10^9^/L	0.83 (0.6, 1.2)	0.7 (0.4, 1.1)	< 0.0001
N/L	9.3 (5.6–17.2)	14.3 (7.6–24.6)	< 0.0001
HgB, g/L	105 (88, 121.5)	105 (89, 123)	0.492
Platelet count, ×10^9^/L	201 (136.5, 287)	177 (113, 251.8)	< 0.0001
TP, g/L	56.3 (51.8, 61.0)	56.0 (50.2, 60.6)	0.111
ALB, g/L	29.9 (27.2, 33.1)	28.8 (25.8, 32.2)	< 0.0001
LDH, U/L	298.5 (234, 424.8)	387 (268, 573)	< 0.0001
CRE, μmol/L	56 (41, 82.5)	67 (48, 114)	< 0.0001
BUN, mmol/L	8.2 (5.5, 12.6)	10.9 (7.0, 17.9)	< 0.0001
CRP, mg/L	84.9 (40.9, 140.1)	107.1 (48.0, 172.8)	< 0.0001
PCT	0.6 (0.2, 2.1)	1.2 (0.3, 4.9)	< 0.0001
Glu, mmol/L	7.1 (5.8, 9.5)	7.3 (5.7, 9.8)	0.685
D-dimmer, μg/mL	2.4 (1.3, 4.8)	3.4 (1.7, 7.1)	< 0.0001
Lac, mmol/L	1.4 (1.1, 1.9)	1.8 (1.4, 2.4)	< 0.0001
VD	159 (22.8%)	410 (64.1%)	< 0.0001

### Selection of predictive variables

**Figure 2:**
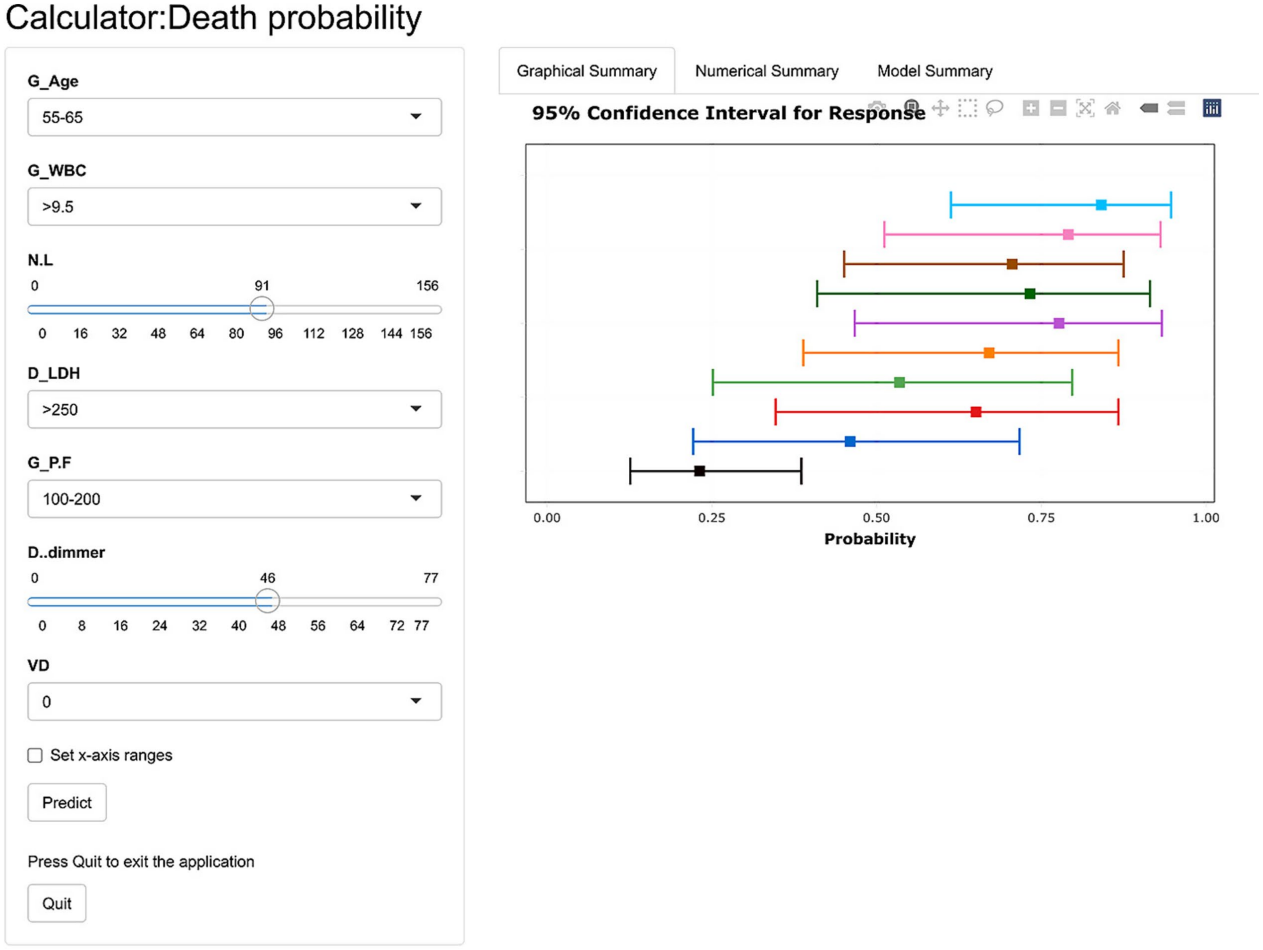
The web-based calculator of the prediction model. The doctors are asked to put integers to the web-based calculator as following: the patient’s age (1: < 45 years; 2: 45–54 years; 3: 55–64 years; 4: > 65 years), PaO2/FiO2 (mmHg 1: < 400; 2: < 300; 3 < 200; 4: < 100), white blood cell count (1: 3.5–9.5 × 109 / L; 2: < 3.5 × 109 / L; 3: ≥ 9.5 × 109 / L) and lactic dehydrogenase level (0; < 250 U / L; 1: > 250 U / L).

The LASSO regression indicated 7 variables independently related to the SSP prognosis, including age, white blood cell count, NLR, lactic dehydrogenase, PaO2/FiO2, D-dimer and vasoactive drug use (Table 3). The prediction model was publicly available as an online calculator^1^ (Figure 2).

**Table 3:** Lasso regression analysis of 28-day mortality in the derivation cohort.

Characteristic	OR	95% CI	*p* value
Age (years)			
1 (≤ 45)	1 (ref)	–	
2 (46–54)	0.79	0.39, 1.61	0.5
3 (55–64)	1.11	0.58, 2.13	0.8
4 (≥ 65)	1.75	1.01, 3.10	0.048
WBC (× 10^9^ / L)			
1 (3.5–9.5)	1 (ref)	–	
2 (≤ 3.5)	1.71	0.76, 3.87	0.2
3 (≥ 9.5)	1.50	1.08, 2.07	0.015
NLR	1.01	1.00, 1.02	0.006
LDH	1.77	1.25, 2.53	0.002
P/F (mmHg)			
1 (≤ 400)	1 (ref)	–	
2 (≤ 300)	0.97	0.59, 1.59	0.9
3 (≤ 200)	1.32	0.82, 2.15	0.3
4 (≤ 100)	2.15	1.21, 3.86	0.010
D-dimmer	1.03	1.01, 1.06	0.008
VD (yes or no)	4.64	3.45, 6.26	< 0.001

### Performance evaluation of the proposed model

**Figure 3:**
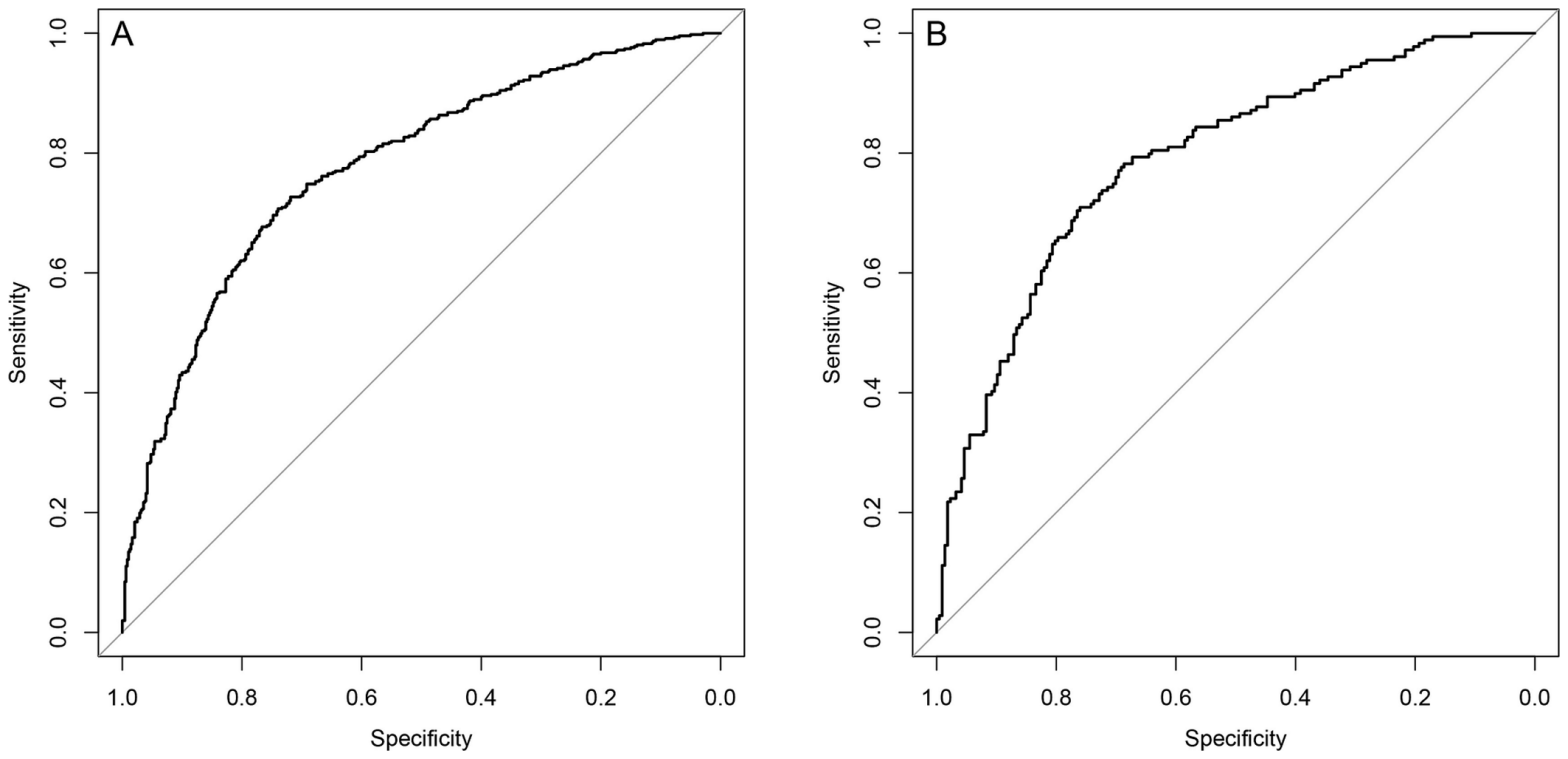
ROC curves for the prediction model. **(A)** The area under the curve of the prediction model in the derivation cohort is 0.777. **(B)** The area under the curve of the prediction model in the validation cohort is 0.803. The Bier score of the model was 0.197 in internal validation and 0.179 in external validation.

The prediction model showed an AUC of 0.777 and 0.803 in the derivation cohort and validation cohort, respectively, indicating a good discrimination power of the model (Figure 3).

**Figure 4:**
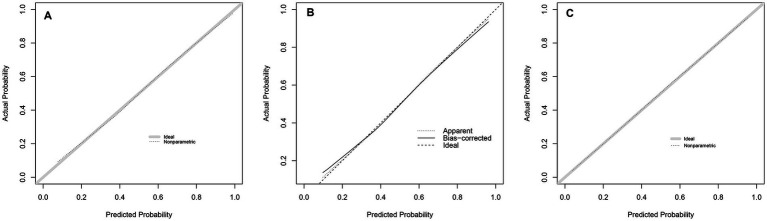
Calibration plot of the prediction model. **(A)** derivation population, **(B)** internal validation, **(C)** external validation populations. In panels **A** and **C**, the solid diagonal line represents the ideal calibration and the dotted line is the calibration line of the prediction model. In panel **B**, dashed diagonal line represents the ideal calibration, straight line represents the bias-corrected calibration line of the prediction model, and dotted line represents the apparent calibration line of the prediction model.

The Bier score of the model was 0.197 in internal validation and 0.179 in external validation. Moreover, the prediction model showed a high overlap between calibration curve and ideal curve in internal validation and external validation, indicating the high calibration of the proposed model (Figure 4).

### Comparison of accuracies between models

**Figure 5:**
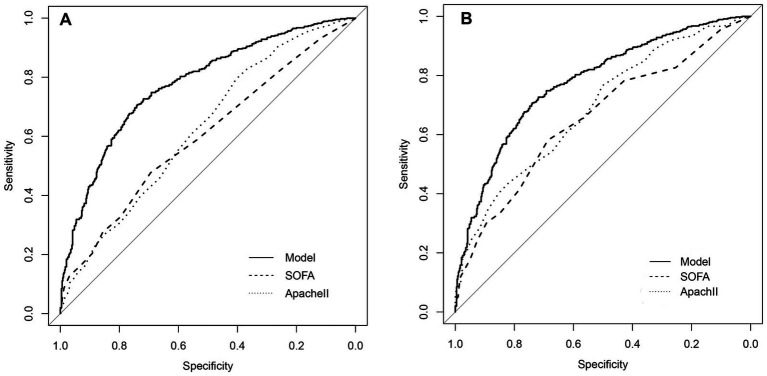
Comparison of discrimination function of the proposed model, SOFA and Apache II in the SSP patients. **(A)** The area under the ROC curve (AUC) of the proposed model, SOFA and Apache II in the derivation cohort is 0.777, 0.600, 0.625, respectively (*p* < 0.05). **(B)** The area under the ROC curve (AUC) of the proposed model, SOFA and Apache II in the validation cohort, is 0.803, 0.655, 0.688, respectively.

The median SOFA score was not significantly different between death group and survival group in the derivation cohort (6 (4, 9) *vs.* 5 (3, 7)) or the validation cohort (5 (5, 10) *vs.* 5 (3, 7)). The APACHE II score in the death group *vs.* survival group was 19 (15, 24) *vs.* 17 (11, 21) in the derivation cohort and 20 (15, 25) *vs.* 14 (10, 20) in the validation cohort. The proposed model had a significantly higher prediction performance than SOFA and APACHE II scores in the derivation cohort (AUCs: 0.777 *vs.* 0.600 *vs.* 0.625, *p* < 0.05). Besides, the proposed model had a significantly higher prediction performance than SOFA and APACHE II scores in the validation cohort (AUCs: 0.803 *vs.* 0.655 *vs.* 0.688, *p* < 0.05) (Figure 5, Table 4).

**Table 4:** The prognostic predictive value of three scoring systems.

Score system	AUC	95%CI	*p* value
Derivation cohort			
SOFA	0.600	0.564–0.6356	*p* < 0.001
Apache II	0.625	0.589–0.660	*p* < 0.001
Proposed model	0.777	0.743–0.802	*p* < 0.001
Validation cohort			
SOFA	0.655	0.601–0.710	*p* < 0.001
Apache II	0.688	0.634–0.740	*p* < 0.001
Proposed model	0.803	0.759–0.846	*p* < 0.001

## Discussion

In this single-center retrospective cohort study, we evaluated the clinical and laboratory characteristics in SSP patients, developed a mortality risk prediction model for SSP patients, and built a web-based calculator of mortality prediction. The model included age, white blood cell count, neutrophil-to-lymphocyte ratio, lactate dehydrogenase, PaO2/FiO2, D-dimer and vasoactive drug use. The proposed model had an AUC of 0.777, indicating a better prediction performance than SOFA score and APACHE II score.

The SOFA score and APCHE II score have been widely utilized for prognosis assessment in pneumonia and sepsis, but study results are discrepancy. In January 2014, the Sepsis-3 working group of Society of Critical Care Medicine and European Society of Intensive Care Medicine recommended a SOFA score ≥ 2 for sepsis diagnosis. However, the SOFA score does not include age or comorbidities and therefore has limited applications in clinical practice. The APACHE II score proposed by Knaus mainly includes age, comorbidities and Glasgow Coma Scale score, leading to the underestimation of disease severity in young patients who are healthy before hospital admission (21). In this study, the AUC of SOFA score and APCHE II score is 0.600 and 0.625, respectively, showing their limited predictive values in the 28-day mortality in SSP patients.

Age is a widely recognized independent predictive factor of mortality in SSP patients. Aging patients, especially those over 65 years, have a weak immune system, poor nutritional status and chronic comorbidities, thus the occurrence and mortality of sepsis are higher following pneumonia onset (22–24). Holter et al. (25) observed a 83% increase of pneumonia mortality risk per 10 years of age during 5-year follow-up. Montull et al. (26) and Kolditz et al. (27) reported age as an independent mortality risk in pneumonia patients. In a multi-center, retrospective study in China, Han also identified age as a risk factor of in-hospital death in pneumonia patients (odds ratio: 1.01, 95% confidence interval: 1.01–21.03), and the mortality rate significantly increases with age (28). Our findings support these studies by showing a significantly higher mean age in death group than survival group. Furthermore, the Logistic regression analysis also confirmed age as an independent risk factor of death in SSP patients and indicated an increasing SSP mortality rate with age.

NLR is an indicator for the severity assessment of systemic infection and inflammatory response by calculating the ratio of neutrophil count over lymphocyte count. In healthy adults, the NLR is between 0.78–3.53 (29). The systemic inflammation caused by pneumonia is mainly characterized by increasing neutrophils and decreasing lymphocytes. Hence, a high NLR indicates a severe inflammatory response. Liu and Hwang reported NLR as a risk factor of poor prognosis in sepsis patients (30, 31). Pantzaris et al. (32) found a close relation between NLR and SSP death. In our study, NLR was significantly higher in death group than survival group in both derivation cohort (12.9 (7.1, 23.6) *vs.* 8.3 (5.4, 15.8), *p* < 0.001) and validation cohort (16.6 (9.6, 29.2) *vs.* 12.1 (6.5, 18.8), *p* < 0.001) in our study. Moreover, the higher is the NLR, the poorer the patients’ prognosis will be. NLR shows the relative change between neutrophil and lymphocyte and has a high predictive value for SSP. Thus, the NLR instead of neutrophil count or lymphocyte count alone should be evaluated clinically.

Vasoactive drugs are required to maintain a mean arterial pressure above 65 mmHg to ensure sufficient tissue perfusion once septic shock is identified in SSP patients (33). The patients complicated by septic shock showed a significantly higher mortality rate (3). In our study, vasoactive drugs were more frequently used in death group than survival group (64.1% *vs.* 22.8%, *p* < 0.05). PaO2/FiO2 ratio was also reported to decrease with the severity of respiratory failure and mortality rate (34–36). In our study, the PaO2/FiO2 ratio was significantly lower in death group than survival group in both derivation cohort (169.8 (109.6, 231.5) mmHg *vs.* 203.3 (154.9, 259.4) mmHg, *p* < 0.05) and validation cohort (181.7 (122.0, 242.3) mmHg *vs.* 223.0 (164.5, 287.2) mmHg, *p* < 0.05). D-dimer elevation has been reported to relate to the outcomes in both pneumonia and sepsis patients, which is also confirmed in this study (37–39).

Our study has several advantages. Firstly, the 7 variables included in our proposed model can be easily acquired in hospital since they are commonly tested at the patients’ admission. The prediction model is also usable for primary hospitals with its noninvasiveness, simplicity and accuracy. Secondly, our proposed model is publicly available as a web-based calculator which can be freely accessible by global doctors for further validation. The clinicians can evaluate the patients’ mortality risk at their hospital admission by using the clinical data so as to tailor the monitoring and treatment plans for the patients to improve their prognosis. Thirdly, our proposed model has been internally and externally validated, and the discrimination and calibration analyses have shown the good predictive performance of the model. These findings provide evidence to support the generalizability of our proposed model.

Some limitations are worthy of consideration in interpreting our study results. Firstly, the study was single-center and retrospective, so some potentially relevant immune indicators such as interleukin – 6 and T cell subpopulations were not included and their relations to SSP severity were not investigated. Secondly, the study had a small sample size, and we did not explore the impacts of different pathogenic microorganisms, antibiotics and lactic acid on prognosis. Thirdly, our study was performed in a tertiary hospital which admits mostly severe patients, thus selection bias might exist in choosing the study population. Lastly, the findings of this retrospective study have not been confirmed in a prospective study, but the study provides some bases for future study design. Overall, a large-sample-size, multi-center prospective study may be needed to further validate our results.

## Conclusion

In conclusion, the web-based mortality risk prediction model is likely to help the clinical decision makers to identify the high-risk SSP patients at an early stage so as to optimize the treatments, improve the patients’ prognosis and reduce the mortality rate.

## Data Availability

The raw data supporting the conclusions of this article will be made available by the authors, without undue reservation.

## Footnotes

^1^ https://yzg1972yzg1972.shinyapps.io/dynnomapp
